# Multiplatform characterization of online permanent female contraception discussion among social media users: analysis of Twitter and Reddit

**DOI:** 10.1186/s12978-025-02239-z

**Published:** 2025-12-23

**Authors:** Tiana J. McMann, Michael R. Haupt, Nicolette Le, Karina Backes-Jedrzejek, Marielle E. Meurice, Zhuoran Li, Tim Ken Mackey

**Affiliations:** 1 Global Health Policy and Data Institute, San Diego, CA USA; 2 S-3 Research, 9150 Chesapeake Drive Ste 290, San Diego, CA 92123 US; 3 Global Health Program, Department of Anthropology, University of California San Diego, 9500 Gilman Drive, #0532, MC: 0505, La Jolla, San Diego, CA 92093-0532 US; 4 Department of Cognitive Science, University of California San Diego, 9500 Gilman Dr, La Jolla, San Diego, CA 92037 US; 5 Division of Complex Family Planning, Department of Obstetrics, Gynecology, and Reproductive Sciences, University of California San Diego, 9300 Campus Point, Mail Code 7433, La Jolla, San Diego, CA 92037-7433 USA

**Keywords:** Permanent female contraception, PFC, Dobbs v. Jackson, Sterilization, Social media, Dobbs v. Jackson

## Abstract

**Objective:**

Individuals choosing permanent female contraception (PFC) face barriers including age and parity. Prior literature has focused on regret, but rarely on understanding patient perspectives. Social media is increasingly used to obtain medical information; hence this study seeks to use popular platforms to evaluate motivations, barriers, and facilitators to obtaining PFC.

**Methods:**

This study collected Twitter and Reddit posts from October 2020 to April 2023 and July 2017 to April 2023, respectively. Data was analyzed using Bidirectional Encoder Representations from Transformers (BERT), followed by manual deductive coding of relevant topic clusters to characterize user-generated PFC discussions. We collected 409,641 posts including 321,267 tweets and 88,374 Reddit posts and performed content analysis using a deductive coding schema using the socio-ecological model approach to determine which posts to include in the final analysis. Sentiment analysis was conducted to detect emotions and themes most correlated with post engagement.

**Results:**

We identified 2,356 posts, including 2,076 tweets (88.12%) and 280 subreddit posts (11.88%) from Reddit relevant to PFC discourse. Major themes included clinician (*n* = 246; 10.44%), patient (*n* = 1,388; 58.91%), interpersonal (*n* = 254; 10.78%), institutional (*n* = 311; 13.20%), and policy-level perspectives (*n* = 157; 6.66%) and derived 22 subthemes. The top subthemes included patients’ seeking/sharing PFC advice (20.80%), discussion of successful completion or commitment/intent to undergo PFC (27.04%), interactions with healthcare providers and beliefs surrounding PFC regret (10.77%), the cost associated with PFC (7.05%), and the Dobbs V. Jackson ruling (6.66%). Additionally, there was a significant increase in posts on PFC following the Dobbs decision. Sentiment analysis shows that posts containing emotional words (both positive and negative) and words related to themes such as home, friends, and family were more likely to receive engagement on Reddit while sentiments related to health, optimism, and communication were correlated with tweet engagement.

**Conclusion:**

As reproductive healthcare continues to face restrictions, online communities provide insight into the motivations and decision-making behaviors of people seeking PFC. Findings can help clinicians better understand patient perspectives, and improve our ability to provide person-centered contraception care for patients desiring PFC.

**Supplementary Information:**

The online version contains supplementary material available at 10.1186/s12978-025-02239-z.

## Introduction

On June 24, 2022, *Dobbs v. Jackson Women’s Health Organization* overturned a nearly 50-year precedent set by the 1973 Supreme Court of the United States (SCOTUS) ruling in *Roe v. Wade* that protected the right to abortion. In the wake of the *Dobbs* decision, there has been a noticeable increase in interest and demand for permanent female contraception (PFC) [1, 2]. This surge was observed both following the SCOTUS leak on May 2, 2022, and the official ruling [2–5]. PFC includes bilateral salpingectomies and tubal ligations and are among the medically available contraceptive options for individuals to meet their personal reproductive goals. According to the most recent National Survey of Family Growth data from 2017 to 2019, PFC is the most common method of contraception among women aged 15–49 in the US, relied on by more than 18% of women, prior to the Dobbs decision [6]. Furthermore, occlusion is one of the top 6 most frequently performed surgeries, behind non-gender-specific surgeries such as cardiovascular surgery and diagnostic and therapeutic procedures [7, 8]. As such, PFC has gained more attention and interest following the rollback of protection for abortion care [2, 4, 5]. 

However, individuals choosing to exercise reproductive autonomy by seeking PFC may also face barriers, including, but not limited to, denial of service [9]. Refusals to provide PFC procedures may stem from the desire to prevent regret in patients under the age of 30, as age is a factor that potentially correlates with relatively higher rates of regret [10]. Physicians have also expressed tension between wanting to achieve balance between respect for patient autonomy and concern for potential future regret, with some worried that patients lack the understanding to make an informed decision [11]. Physician counseling surrounding PFC has also been shown to be limited, with discourse showing frequent discouragement of such methods when discussed [12]. 

Though many studies focus on the topic of regret, few seek to understand the overwhelming majority of patients who did not face regret, their motivations for PFC, and their barriers and facilitators to reproductive care [13]. This may be even more pertinent as an increased number of individuals seek PFC options which may be reflective of the fear of losing full reproductive autonomy secondary to abortion restrictions [1]. Several potential emerging problems have been identified as women seek PFC provision post-*Dobbs* including discrimination, accessibility, and institutional policies [1, 14]. However, no studies to date have evaluated these emerging challenges and potential motivators, particularly in the context of online discussions.

Social media platforms have emerged as trusted sources of health information and represent access to important online communities that actively engage in discourse on sensitive health-related issues, including contraception [15–18]. However, to our knowledge, no studies have utilized social media to analyze issues regarding PFC. Hence this study aimed to conduct a multiplatform social listening study to assess potential motivations, facilitators, general discussion, and barriers to PFC with the aims of providing diverse perspectives to the current and evolving provision of reproductive care. Twitter and Reddit are social media platforms which allow users to engage in politicized and non-political conversations with others and outsource information [16, 19]. While Twitter, with over 330 million monthly users, provides a platform for succinct posts, Reddit, the sixth most accessed website in the US, provides longform, data rich conversations [19, 20]. We also assessed which types of sentiments and themes are most likely to evoke engagement within the PFC discourse on Twitter and Reddit as posts with high engagement are promoted more often in newsfeed algorithms and can reflect views that are either convergent or divergent from community norms [21]. 

## Methods

### Data collection

Data were collected from Twitter (X) and specific subreddits from Reddit using the respective platform’s application programming interface (API) (e.g., the then available public streaming API for Twitter and PushShift API for Reddit). Keywords associated with sterilization procedures (#bisalp #salpingectomy #sterilization) were used to collect data from Twitter retrospectively from October 2020 to April 2023 and all posts from subreddits r/sterilization and r/childfree from Reddit were collected spanning July 2017 to April 2023. R/sterilization is a forum specifically for users to discuss PFC with an estimated 9,800 members while r/childfree is a forum for discussions by individuals who do not have and never want to have children with an estimated 1.5 million members at the time of data collection. Study keywords were selected based on initial manual searches on X and Reddit for relevant content to find discussion related to the topic of interest as well as Internet user search trend analysis using the keywords without the use of the hashtag (#) on Google Trends over the same time. We pursued a multiplatform study to analyze a variety of user-generated discussions from diverse web-based communities and to assess different online communication and interaction on the topic [22]. 

For data collected from the two subreddits, each post was ranked based on engagement (i.e., upvote and comments) and the top 150 posts were selected for content coding. On Reddit, upvoting generally reflects content that is perceived as useful and trustworthy among users (see Supplement for additional information) [23]. 

### Topic modeling

To characterize and elucidate decision-making processes, experiences, and barriers associated with individuals seeking PFC, we used a form of Natural Language Processing (NLP) to identify and categorize topics and word groupings relevant to the study objectives into thematic clusters and outliers. We used Bidirectional Encoder Representations from Transformers (BERT), which is a pre-trained, self-supervised transformer-based algorithm, which evaluates each word in a phrase bidirectionally, providing deep contextual understanding. BERT then produces an output of data which includes the sum of the posts into a determined number of *k* (k = 20) clusters based on the posts’ semantic similarities or relation. The remainder of tweets which are not semantically similar are considered “outliers” by BERT. BERT was selected due to its use in previous work that analyzed large-scale social media data, including for reproductive healthcare topics [18, 24–26]. The study used BERTopic Version 0.6.0 with Python Version 3.7.

Using BERT, we identified topic clusters with word groupings, frequencies, and characteristics that appeared to be related to user-generated conversations associated with PFC (*signal clusters*) and then extracted all posts from these topic clusters for manual annotation. Two topic clusters were selected after initial screening, resulting in 10,396 tweets. Tweets were further screened based on engagement through post engagement, resulting in 2,914 selected for manual content coding for relevance to PFC-related topics.

### Content analysis

Twitter posts selected by using BERT and all Reddit posts were manually reviewed for in-depth content analysis. Posts were considered “signal” posts if they were: user generated (i.e., posts from individual users discussing their opinions, beliefs, and experiences with PFC) and discussed PFC procedures including tubal ligation, sterilization, bilateral salpingectomy, and other PFC-related topics or terms (e.g., bisalp, tubes tied, sterilized, occlusion, hysterectomy). Posts related to news or media coverage about PFC, organizational or healthcare institution posts, and posts not related to sterilization or PFC (e.g., hospital sterilization of surgical instruments or vasectomy) were excluded from further analysis.

A general deductive coding schema using the socio-ecological model (SEM) that outlines the interrelation of health and behavior through various levels including the “Individual”, “Interpersonal”, “Institutional”, and “Policy”, were selected as parent codes as it has been used in a prior women’s reproductive healthcare research including contraception use [27]. Facilitators are operationally defined in this study as a variety of factors which improve knowledge, access or utilization of PFC-related care, while barriers are a lack thereof and are various factors which provide obstacles to desired PFC care [27]. All posts were reviewed by first through fourth authors, and notes were taken on general themes of posts from which an initial codebook was created. SEM categories of PFC-related discussions were created throughout our deductive coding. First through fourth authors achieved a high intercoder reliability (Cohen *κ* = 0.95). Discrepancies in coding were discussed amongst authors and consensus on correct classification was reached. For sentiment analysis see Supplement.

## Results

**Fig. 1:**
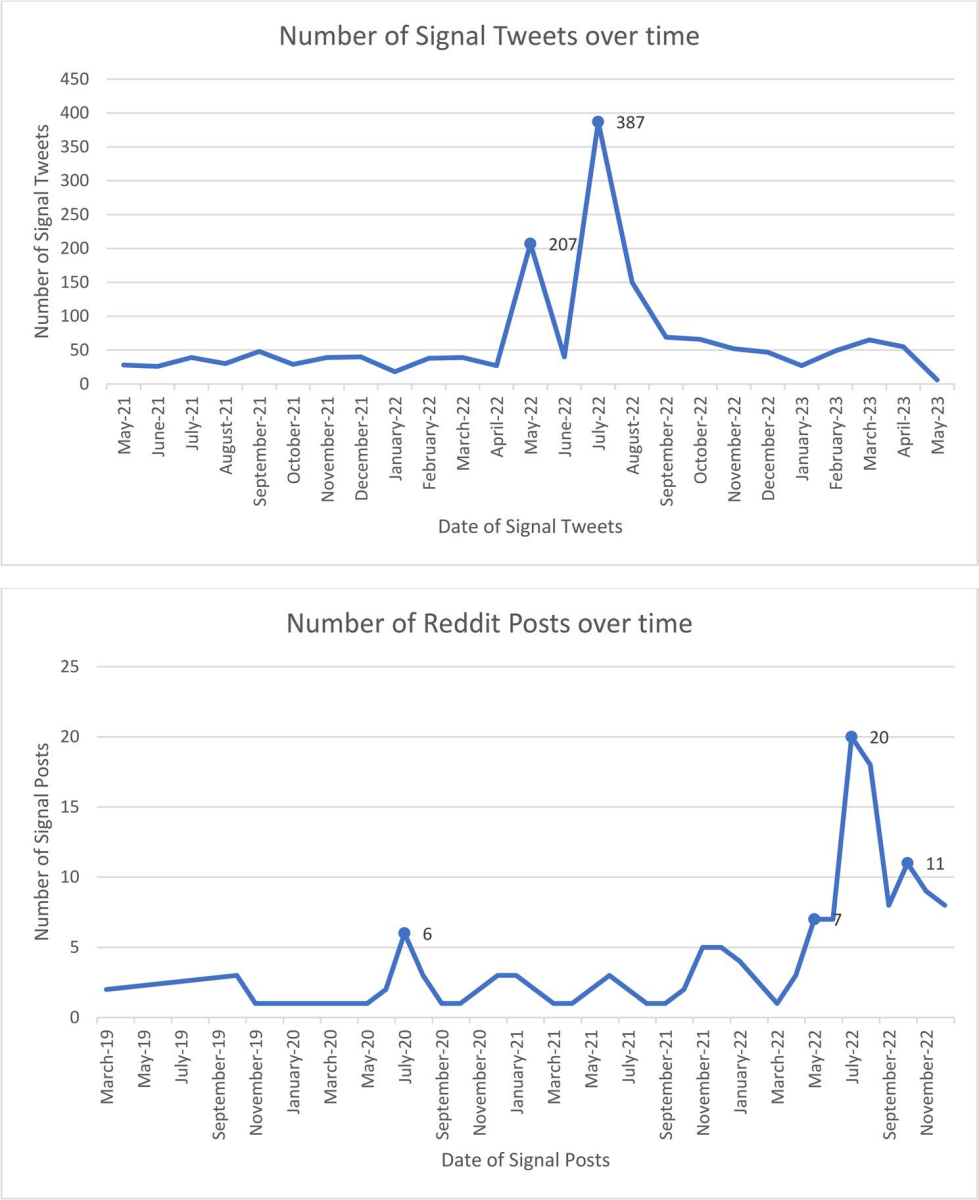
Total Twitter and Reddit signal tweets over time of study with spikes in discussion in May and June 2022

A total of 409,641 posts were collected from X and Reddit using PFC-related keywords and selected subreddits and included data spanning from July 2017 to April 2023 based on the method and availability of data collection used. The dataset comprised of 321,267 (78.43%) unique tweets and 88,374 (21.57%) Reddit posts. After applying BERT, engagement score filtering, and manual annotation, 3,216 posts (2,914 from Twitter and 302 from Reddit) were included for further analysis of which 2,356 (73.26%) were identified as associated with PFC-related topics (2,076 from Twitter (88.12%) and 280 from Reddit (11.88%)). The period covered by this subset of signal posts was from June 2019 (earliest posted on Reddit) to April 2023 (latest posted on X). We found an increase in discussion related to PFC-related topics on both Twitter and Reddit around the time of the SCOTUS leak as well as the official decision to overturn *Roe v. Wade* (refer to Fig. 1a and b for number of posts over time). Between May and July 2022, dates central to the SCOTUS leak and decision, 634 signal tweets and 34 Reddit posts were detected related to PFC discussion.

### Content analysis

Our qualitative analysis and deductive coding approach derived 22 topics based on the SEM adaption (see Table 1 for a complete breakdown of the stratification of these codes and subcodes for each social media platform). Following the SEM framework, all detected topics were first classified into 5 parent domains: individual, into which we differentiated individual clinician (239/2,356, 10.14%) and individual non-clinician (1,395/2,356, 59.21%), interpersonal (254/2,356, 10.78%), institutional (311/2,356, 13.20%), and policy (157/2,356, 6.66%) (refer to Table 1 for codes and subcodes and deidentified examples).

**Table 1:** Stratification of permanent female contraception parent domain and subcodes topics detected on Twitter and Reddit

			Twitter		Reddit	
System	Code	Description	% by platform	Count	% by platform	Count
Individual (Clinician)	A1a	Intent to not provide procedure	0	0	0	0
	A1b	Intent to provide/has provided proceed	6.1	99	0.7	1
	A1c	Advice seeking/sharing	7.2	113	0	0
	A1d	Anecdotal reference to increases in patients seeking procedure	1.8	26	0	0
Individual (Non-Clinician)	A2a	Intent to not receive procedure	0.4	7	0.7	1
	A2b	Intent to receive	13.8	22	12.5	19
	A2c	Commitment to receive (appointments/consultations scheduled)	11.8	195	40.1	61
	A2d	Completion of procedure	20.4	333	34.2	52
	A2e	Advice/information seeking/sharing	26	422	44.7	68
	A2f	General discussion/reactionary (non-informative)	13.3	215	0	0
Interpersonal	B1	Negative interaction with HCW	2.3	38	5.3	8
	B2	Positive interaction with HCW	3.6	58	10.5	16
	B3	Denied procedure	2.5	39	7.2	9
	B3b	due to “regret risk” (Age/child-bearing-related)	0.7	12	3.9	6
	B3c	marital status	0.1	1	0	0
	B3d	doctor belief	0.2	3	2	3
	B4	Sharing information/discussion related to “regret risk” (age-related)	3.6	59	1.3	2
Institutional	C1	Cost/insurance/financial discussion	9.4	153	8.6	13
	C1a	Insurance/cost obstacle (denied, unexpected costs, not having insurance)	3.4	55	3.9	6
	C1b	Insurance/cost facilitator (preauthorized, covered procedure)	4.1	66	4.6	7
	C2	Difficulty obtaining procedure	0.7	11	0	0
Policy	D1	Dobbs v. Jackson decision/Roe v. Wade overturned	9.2	149	5.3	8

**Fig. 2:**
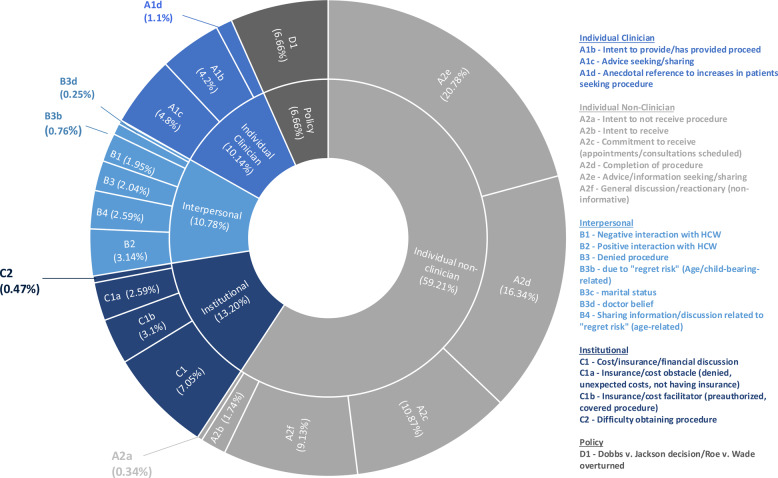
Total Twitter and Reddit volume-based distribution of permanent female contraception parent domain and subcode topics

Posts identified in the individual clinician domain categorized discussions by users who self-identified as a clinician through explicit text within the post (see Tables 1 and 2 for examples of clinician-based discussion). Discussions within this parent domain focused on a clinician’s intent to provide or having provided a PFC procedure (A1b; *n* = 100, 4.2%) (See Fig. 2 for distribution of data across the various parent domains and subcodes), clinicians seeking or sharing PFC provision-related advice (A1c; *n* = 113, 4.80%), and discussions related to increased number of patients seeking PFC options (A1d; *n* = 26, 1.1%). Among our 22 deductive codes, only one topic yielded no results, specifically, intent to not provide a procedure within the individual clinician domain (A1a).

**Table 2:** Stratification of permanent female contraception subcode topics detected on Twitter and Reddit (including de-identified and paraphrased examples)

Code	Description	Example Tweet
A1a	Intent to not provide procedure	
A1b	Intent to provide/has provided proceed	That giant smile on a child free patient’s face when I tell them I’ll put in the surgery request for sterilization with no second guessing them or pushback. I’m here to help you
A1c	Advice seeking/sharing	If you’re someone with a uterus who is considering sterilization, here is your friendly gyn/onc genetic counselor reminder that a bilateral salpingectomy may reduce your risk for ovarian cancer, in addition to preventing pregnancy.
A1d	Anecdotal reference to increases in patients seeking procedure	I’ve had 4 humans bearing a uterus ask me if I would do their salpingectomy since Friday (this is in the setting of no clinic/on vacation). Women are worried about their bodily autonomy. My answer was “yes” to all (all heard my spiel about not reversible/regret)
A2a	Intent to not receive procedure	Having children is not in the cards for me. I don’t want them, nor does my partner and that’s okay! After a short discussion my partner decided to get a vasectomy so I wouldn’t have to go through the sterilization surgery. We are a team and this was a team decision
A2b	Intent to receive	Becoming pregnant would be a life threatening health issue for me so this ruling has been one of my most real fears come to life today. Im setting aside as much money as I can for surgical sterilization.
A2c	Commitment to receive (appointments/consultations scheduled)	Signing my sterilization consent forms
A2d	Completion of procedure	if anyone with a uterus is looking to get sterilized and can get to the harrisburgish area of PA, I know a doctor who will do it! he did my bisalp when I was 22 and was great about it. just dm me and I’ll give you his name
A2e	Advice/information seeking/sharing	Here is a list of over 150 doctors willing to preform tubal sterilization no questions asked. Please share if you can.
A2f	General discussion/reactionary (non-informative)	any doctor who refuses sterilization because “you’re too young to decide that, you’ll change your mind” I’m going to spit in your face
B1	Negative interaction with HCW	Woke up mad at all the times my obgyn denied me sterilization. If I were to get pregnant, it would be very bad due to all my medical issues
B2	Positive interaction with HCW	Today my new gyno offered me the option of sterilization. it felt great to even have that option be discussed with me instead of me being told I *might* change my mind. I wish that more doctors discussed the full array of birth control.
B3	Denied procedure	My husband and I have both been refused sterilization procedures. We’re now both over 45, no biological kids.
B3b	due to “regret risk” (Age/child-bearing-related)	When I scheduled a consult for surgical sterilization, they misunderstood and put me with the NP. She tried to convince me to try other methods like an IUD first, you know, because I couldn’t possibly know what I wanted? There are other options. I’m young. Might change my mind.
B3c	marital status	One doctor refused, saying someday the patient might have a husband who wants kids. Another issued comments such as, “So your mom is never going to be a grandma?”
B3d	doctor belief	it took 11 years of begging, searching, moving states to get a bilateral salpingectomy. it took multiple appointments and refusing to back down to get an IUD rather than another pill.
B4	Sharing information/discussion related to “regret risk” (age-related)	There is currently a formula shortage. We have no universal healthcare. We have no real paid maternity leave. We have no comprehensive sex education. You are shamed for buying condoms or accessing other forms of birth control. Women under a certain age are denied sterilization.
C1	Cost/insurance/financial discussion	Reminder that most insurances will cover female sterilization once u hit 21 and Planned Parenthood has clinics that do them on site or they will direct to a place that does do it
C1a	Insurance/cost obstacle (denied, unexpected costs, not having insurance)	I was today old when I found out that Medicaid requires 30 day waiting period, and lots of doctors appointments for a woman to obtain sterilization surgery. Give it a think. Medicaid covers a disproportionate amount of low income & disabled women.
C1b	Insurance/cost facilitator (preauthorized, covered procedure)	My insurance covered my surgical sterilization at 100%… Except for the anesthesia. As if that was an optional ad on?
C2	Difficulty obtaining procedure	Also, I heard back about sterilization. I’d have to go through 3 different people and then they decide if I can have it which my psychologist said the likelihood of that happening is extremely rare. Gynecologist, psychologist and even a social worker, and they have to decide.
D1	Dobbs v. Jackson decision/Roe v. Wade overturned	Kentucky clinic claims record surgical sterilization requests from women after SCOTUS ruling A Kentucky-based gynecologist who says her clinic received 91 different requests in just one day from women who want to undergo surgical sterilization.

Within the individual non-clinician domain across X and Reddit, many users posted discussions related to their varying level of commitment and decision-making to undergo PFC including having no intent to receive PFC (A2a; *n* = 8, 0.34%). From here, users discussed their various commitment and how actively they are seeking PFC ranging from an intent to receive PFC (A2b; *n* = 41, 1.74%) which was categorized as users sharing their general interest in receiving PFC and seeking out options related to providers, planning for a future procedure, and how the Dobbs decision has encouraged them to seek PFC. Differentiating from intent to receive PFC were users’ discussions about their commitment to receive PFC (A2c; *n* = 256, 10.70%), a step beyond intent, which was defined as posts discussing their scheduled procedure or consultations but not having received PFC. Further, users also discussed having a PFC procedure (A2d; *n* = 385, 16.34%) in which users posted discussions related to the type of PFC procedure that was performed, their timeline, and their decision-making process. This category generated the most discussion amongst users sharing their level of commitment to PFC.

Individuals also sought and shared PFC-related advice (A2e; *n* = 490, 20.80%) which generated the most discussion within the subcodes of this study. Advice sought or shared on X and Reddit ranged from sharing a list of clinicians who provide PFC, inquiring about post-operative symptoms, and the out-of-pocket costs associated with PFC. Sharing information, particularly a list of clinicians known to have provided PFC amidst denials can improve access to related health services and minimize unnecessary time and additional obstacles. Additionally, lack of knowledge has been identified as a key barrier to contraception use in previous studies [27]. This domain also contained reactionary, non-informative discussions (A2f; *n* = 215, 9.13%), where users shared thoughts, attitudes, and opinions primarily related to the ongoing and recent impacts on PFC and clinician refusal of PFC services, as well as anecdotal references to the SCOTUS decision and how this impacts reproductive autonomy at large.

In the interpersonal domain, which focuses on the interactions between users seeking PFC and any additional individual involved in the process, the highest number of subcodes were detected. This domain included users sharing both positive (B1; *n* = 46, 1.95%) and negative interactions (B2; *n* = 74, 3.14%) with a clinician with context as to how this either facilitated the PFC experience or caused delays in care. Positive interactions included having a streamlined experience when seeking and obtaining PFC while negative interactions were characterized by clinicians presenting external obstacles (e.g., multiple appointments required) to patients seeking PFC. The interpersonal domain also included clinician denial of PFC without a stated reason within the post (B3; *n* = 48, 2.04%), denial due to age (B3b; *n* = 18, 0.76%), due to marital status (e.g., not being married and seeking PFC) (B3c; *n* = 1, 0.04%), and due to clinicians’ personal belief against providing the procedure (B3d; *n* = 6, 0.25%). Within this domain, we also detected general discussion related to risk of regret as a barrier to PFC (B4; *n* = 61, 2.59%). This subcode was characterized by non-personal or anecdotal references to clinicians denying or obstructing PFC for patients, a direct obstacle to an individual receiving desired health services.

The institutional domain focused on discussions related to organizational influence on users seeking PFC, including hospital or insurance-related policies. We found general discussion of users sharing information regarding insurance or cost-associated facilitators or barriers to PFC without reference to personal experiences (C1; *n* = 166, 7.05%), while also detecting explicit individual encounters with insurance or financial obstacles (C1a; *n* = 61, 2.59%) and facilitators (C1b; *n* = 73, 3.10%). This includes specific insurance coverage required to meet certain eligibility criteria for PFC, such as a mandated waiting periods, or not covering the procedure, or covering large portions of the out-of-pocket cost of PFC. Finally, we also found general institutional-related discussion regarding various difficulties or obstacles to PFC due to inexplicit hospital or clinic policy (C2; *n* = 11, 0.47%) (e.g., religious affiliation).

The final SEM domain focused on policy in which the only subcode detected was the *Dobbs* ruling (D1; *n* = 157, 6.66%). This theme was prominent throughout posts and included user’s expressing that the *Dobbs* decision was a key factor in seeking PFC as well as expressing direct difficulty, increased barriers, or intensified uncertainty in seeking PFC due to this ruling, as well as information regarding what the ruling is, its scope, and ramifications.

For results on sentiment analysis see Supplement.

## Discussion

To our knowledge, this study is the first multiplatform social listening study to assess PFC motivations and barriers. We collected and analyzed over 409,641 posts across X and Reddit using a combination of NLP, engagement filtering, and content analysis using manual annotation spanning from July 2017-April 2023. This consisted of a large proportion of online conversations originating from X (78.43%). Discussions on the topic revealed numerous themes within 5 SEM-related parent domains: individual clinician (10.14%), individual non-clinician (59.21%), interpersonal (10.78%), institutional (13.20%), and policy (6.66%). While the majority of discussion was generated from X (88.12%), among the two subreddits, r/sterilization resulted in a greater proportion of PFC-related content. The parent domain which generated the most discussion was the “individual non-clinician” domain indicating there were a large number of individual discussions from patients or prospective patients related to PFC. Within this domain, the majority of subcodes detected were individuals sharing or seeking PFC-related advice, as well as sharing they obtained PFC and offering information to users who were interested in seeking PFC.

Social network sites have been well-established as an information source for decision making regarding contraceptives, primarily hormonal birth control and long-acting reversible contraceptives [16]. Findings from this study provide initial evidence that PFC decision-making can also be social and online in nature. In fact, the most prominent theme across both X and Reddit was seeking or sharing PFC-related advice and knowledge (A2e) within the individual non-clinician domain. The type of information users gave or sought differed by platform, with one discussions thread converging on sharing a list of clinicians known to have provided a streamlined or positive experience for those seeking PFC. Hence, women who are considering PFC or have been previously denied may use social networks to find a clinician who may perform the procedure. Sharing this type of information can improve access to specialty care, which is one of the many challenges individuals face when seeking a clinician’s approval for PFC, as found in this study [7]. Furthermore, proactively sharing a list of clinicians may reduce barriers, promote reproductive health equity and autonomy, provide community support, and empower patients [28]. 

We also found themes which may support changing attitudes regarding PFC [10]. Users on both platforms shared general discussion and anger about anecdotal references to women being denied PFC due to their age, or related marital status within the interpersonal domain, both of which stem from what was viewed as paternalistic beliefs. In addition to the general discussion on this topic, users also shared personal experiences of being denied PFC due to these same reasons. Recent discussion around denials to PFC may be a direct cause of women seeking to practice reproductive autonomy but who simultaneously face limited options or general outrage that reproductive options for women continue to be subjugated, particularly in the US [1, 29]. 

Subcodes within the parent domains revealed discussion related to barriers and facilitators and general information related to PFC, including how the *Dobbs* decision has influenced users’ decision-making. Previous studies have reported barriers such as logistical, financial, or clinician-based barriers [30]. This study adds to this knowledge base and helps contextualizes contemporary challenges and facilitators for obtaining PFC in the current reproductive healthcare landscape [29]. This study found negative interactions with clinicians, economical or insurance related barriers, and the *Dobbs v. Jackson* decision as major barriers to PFC, in addition to risk of regret beliefs. Facilitators for PFC included positive clinician interactions, requesting and sharing information online, and adequate insurance coverage. Importantly, the Dobbs decision may also have facilitated increased discussions regarding family planning topics.

Results from this study have implications for clinicians. Clinicians are at the interface of many of the barriers and facilitators related to PFC including interpersonal interactions and insurance coverage decisions. However, they are less likely to be included in social media conversations that are taking place outside of the office, yet at the same time are impacting people’s experience of undergoing PFC. The American College of Obstetricians and Gynecologists (ACOG) recommends discussing regret but posits that age and parity should not be a barrier to PFC [31]. With increasing requests for sterilization following the *Dobb’s* decision and changing attitudes regarding childbearing, clinicians are likely seeing more individuals seeking PFC at younger ages [29]. Being aware of the impact of social media discourse and the types of conversations that are occurring may be helpful for clinicians during their counseling. Additionally, this study provides insight into the patient perspective and motivations for seeking PFC, which is helpful for contemplating strategies focused on person-centered care [32, 33]. 

Findings from this study also show that there is a public interest in information about PFC and family planning topics, and for many, social media platforms are an important source of information, particularly following policy decisions like *Dobbs*. In response, organizations such as ACOG and relevant healthcare institutions should be more proactive with making information available to the general public about PFC. While text-based articles featured on websites can be an effective approach for making clinically-approved information available, other modes of communication such as video content, where health officials discuss PFC topics in a more conversational style, or testimonials that feature a diverse array of people’s personal experiences with PFC, can make this information more accessible to larger segments of the population, could also be promoted on social media to encourage greater reproductive choice and autonomy, and is more effective in disseminating health information [34]. Additionally, for text dominant social media platforms, appropriate reproductive health messaging should be developed to align with recent concerns.

### Limitations

This study has certain limitations. First, it only evaluates data from Twitter and Reddit and was limited in analysis to English tweets, which is not representative of general social media PFC-related discourse, including those occurring on other platforms such as Facebook, TikTok, and Instagram or in Spanish language, the second most commonly spoken language in the US and one of the top languages spoken on Twitter, including specifically those from users who reside in the US but do not speak English and have disproportionate burden of lack of healthcare access [35]. Further, this study only analyzed tweets and Reddit posts posted by users and not comments or other interactions between Twitter and Reddit users in response to a post, which could have yielded additional barrier and facilitator discussion. Importantly, the methodology used between platforms differed in both data collection and data analysis due to the nature of each platform and hence may skew the results. This study likely underreports the total amount of PFC-related content within the dataset since we only coded posts that had a baseline level of engagement. This approach streamlines coding and allow more efficient detection of relevant, user-generated conversations. Additionally, this topic may have also yielded heightened discussion in response to other political events encompassed within this timeframe, potentially increasing the volume of discussions detected in this study.

## Supplementary Information

Supplementary Material 1.

## Data Availability

The dataset supporting the conclusions of this article will be made available upon reasonable request to the senior author for a specified reason.
