# Supplementary material for: Multiplatform characterization of online permanent female contraception discussion among social media users: analysis of Twitter and Reddit
Source: Reprod Health. 2025 Dec 23;23:17. doi: 10.1186/s12978-025-02239-z (PMC12836916; doi:10.1186/s12978-025-02239-z)
Supplement: Supplementary file 1 — Supplementary Material 1. [file 12978_2025_2239_MOESM1_ESM.docx]

1. **Methods**

1.1 Ethics Approval

IRB approval was not required as all information collected from this study was from the public domain and the study did not involve any interaction with users. Any user identifiable information was removed from the study results.

1.2 Data Collection

Posts with high engagement are also promoted more often in newsfeed algorithms and can reflect views that are either convergent or divergent from community norms.^17^ Post engagement was calculated by summing the number of upvotes and comments received for each post. Since comments are considered more active forms of content engagement, comments were weighed twice as much as upvotes as implemented in other social media research.^18–21^

1.3 Sentiment Analysis

Sentiment Analysis

To assess how emotional content influences post engagement, sentiment scores were calculated for all posts collected from X and Reddit. Empath was chosen for sentiment analysis due to its previous use in research characterizing health-related online discourse.^27–29^

Sentiment analysis was conducted on all Reddit posts collected from r/sterilization and r/childfree during the study timeframe and all X posts from the selected topics after applying BERT. Due to the lack of a normal distribution of sentiment scores, we used Spearman’s rho to assess correlations between sentiment scores and post engagement. Engagement behaviors examined for Reddit posts include upvoting and commenting. For X posts, all engagement behaviors (number of likes, retweets, quotes, and replies) were summed together to create an interactive score.

Sentiment scores reflect the percentage of words within a post that correspond to a given sentiment category. Scores are calculated by first counting the number of words associated with a sentiment category and then dividing that count by the total number of words within the post.^25^ Each sentiment category includes a dictionary, which is the list of words selected to represent the category. Sentiment dictionaries can measure a wide array of topics, such as emotional affect states (e.g., anger, fear, joy), biological processes (e.g., health, death), and general topic areas (e.g., money, work, children).^26^ Assigning words to sentiment dictionaries can be accomplished using data-driven approaches, as shown by Stanford’s Empath project that derived over 200 classification categories from analyzing more than 1.8 billion words of modern fiction.

1. **Results**

2.1 Sentiment Analysis

The top 10 sentiments correlated with engagement (upvote and comment) for both r/sterilization and r/childfree subreddits are detailed in **Table 2**. All reported correlations were statistically significant at p<.001. For r/sterilization, posts with higher percentage of words related to *optimism* (r=.29), *positive emotion* (.27), *home* (.27), *negative emotion* (.27), and *nervousness* (.26) were more likely to be upvoted. Other top sentiments correlated with upvoting include *traveling* (.26), *body* (.25), and *domestic work* (.24). Similar to upvoting, *negative emotion* (.13) and *nervousness* (.13) were correlated with commenting. Other sentiments correlated with commenting include *friends* (.13), *children* (.12), *heroic* (.10), *trust* (.10), *timidity* (.09), and *anticipation* (.09). Sentiments related to *health* and *healing* were also top sentiments associated with both upvoting and commenting.

In r/childfree, the top correlated sentiments for both upvoting and commenting were *children* (upvote_u_ = .43, comment_c_ = .41), *family* (_u_ = .40_, c_ = .37), *negative emotion* (_u_ = .38_, c_ = .38), *speaking* (_u_ = .36_, c_ = ..36), *youth* (_u_ = .35_, c_ = .34), *positive emotion* (_u_ = .35_, c_ = .33), *home* (_u_ = .33_, c_ = .29), *communication* (_u_ = .33_, c_ = .33), and *friends* (_u_ = .31_, c_ = .31). Overall, there was more overlap in top sentiments associated with upvoting and commenting within r/childfree (9 out of 10) compared to r/sterilization (4 out of 10). The correlations between sentiment scores were also stronger in r/childfree (range between .29 to.42) compared to r/sterilization (.09 to .29), which may be driven by differences in total number of posts and number of subscribed members within each subreddit.

**Table 3** shows top correlations between sentiment and interactive scores of tweets. Tweets that contain words related to *health* (.09), *optimism* (.09), *meeting* (.08), *communication* (.08), *speaking* (.08), and *attractive* (.08) were most likely to receive engagement. Compared to Reddit posts, correlation strength between sentiment and tweet engagement is consistently weaker.

**Table 2.** Reddit (r/Sterilization and r/Childfree) - Top 10 sentiment correlations with upvote and comment scores, all correlations are statistically significant (p<.001)

| r/Sterilization  (n=1,258 posts) | | | | r/Childfree  (n=87,115 posts) | | | |
| --- | --- | --- | --- | --- | --- | --- | --- |
| *Upvote* | | ***Comment*** | | ***Upvote*** | | ***Comment*** | |
| *Sentiment*  *Category* | *Correlation* | *Sentiment*  *Category* | *Correlation* | *Sentiment*  *Category* | *Correlation* | *Sentiment*  *Category* | *Correlation* |
| optimism | 0.29 | negative emotion | 0.13 | children | 0.43 | children | 0.41 |
| positive emotion | 0.27 | nervousness | 0.13 | family | 0.40 | negative emotion | 0.38 |
| home | 0.27 | friends | 0.13 | negative emotion | 0.38 | family | 0.37 |
| negative emotion | 0.27 | children | 0.12 | speaking | 0.36 | speaking | 0.36 |
| nervousness | 0.26 | heroic | 0.10 | youth | 0.35 | youth | 0.34 |
| traveling | 0.26 | trust | 0.10 | positive emotion | 0.35 | positive emotion | 0.33 |
| health | 0.25 | health | 0.09 | home | 0.33 | communication | 0.33 |
| body | 0.25 | healing | 0.09 | communication | 0.33 | friends | 0.31 |
| healing | 0.24 | timidity | 0.09 | friends | 0.31 | home | 0.29 |
| domestic work | 0.24 | anticipation | 0.09 | party | 0.30 | trust | 0.29 |

*Note*: All correlations are statistically significant at p<.001

**Table 3.** Twitter (n=10,396 posts) – Top 10 sentiment correlations with interactive score (like + retweet + reply + quote), all correlations are statistically significant (p<.001)

| *Sentiment* | Correlation -Interactive  Score |
| --- | --- |
| *health* | 0.09 |
| *optimism* | 0.09 |
| *meeting* | 0.08 |
| *communication* | 0.08 |
| *speaking* | 0.08 |
| *attractive* | 0.08 |
| *celebration* | 0.07 |
| *sadness* | 0.07 |
| *hipster* | 0.07 |
| *childish* | 0.07 |

*Note*: All correlations are statistically significant at p<.001

1. **Discussion**

Sentiment analysis was also conducted to examine emotions and general themes that evoke user participation within PFC discourse. Consistent with previous studies on social media engagement, Reddit posts that contain words reflecting emotional affect (both positive and negative) were positively correlated with engagement in both r/sterilization and r/childfree (p<.001).^33–35^ Other sentiments that were correlated with higher engagement in both subreddits correspond to family planning themes such as *home*, *friends*, and *children*. However, there were also differences in top correlated sentiments that likely reflect distinctions in discourse priorities and users bases between the two subreddits. Post engagement in r/sterilization was more associated with containing words related to *nervousness* (e.g., words such as ‘worried’), *anticipation* (‘unsettle’), *health* (‘suffer’), *heroism* (‘bravery’), and *healing (‘*surgery’*)*, which would correspond to the higher emphasis on PFC discourse and discussions on surgical procedures. However, r/childfree was associated with general themes such as *family (‘*fiancé’*)*, *communication (‘*consult’*)*, *youth (‘*young’*)* and *party (‘*celebration’*)*. On X, engagement with PFC-related tweets was associated with *health* and *optimism* sentiments*,* and sentiments related to coordination such as *meeting, communication*, and *speaking.* In contrast to Reddit, *nervousness* and *negative emotion* were not top correlated sentiments, suggesting that PFC-related discourse was more positive on X. Differences in how platforms address psychological needs among users (e.g., seeking information vs support) may further impact how users make decisions surrounding PFC.
